# P-267. Predictors of mortality among adult PLHIV receiving antiretroviral therapy in AIDS centers in Sughd Province, Tajikistan: a retrospective cohort study

**DOI:** 10.1093/ofid/ofaf695.488

**Published:** 2026-01-11

**Authors:** Emomali Qurbonov, Roberta Horth, Aisuluu Kubatova, Dilyara Nabirova

**Affiliations:** Central Asia Advanced Field Epidemiology Training Program, Khujand, Sughd, Tajikistan; US Centers for Disease Control and Prevention, Dulles, Virginia; Ministry of Health of the Kyrgyz Republic, National Institute of Public Health, Bishkek, Kyrgyzstan, Bishkek, Bishkek, Kyrgyzstan; CDC Central Asia office, Almaty, Almaty, Kazakhstan

## Abstract

**Background:**

Despite significant advances in antiretroviral therapy (ART), HIV-related mortality remains an important global health problem in low-income countries, including the Sughd region of Tajikistan (Figure 1). Our study aimed to assess mortality rates among people living with HIV (PLHIV) on ART and to identify key factors influencing mortality in Sughd, Tajikistan.

**Fig. 1.**
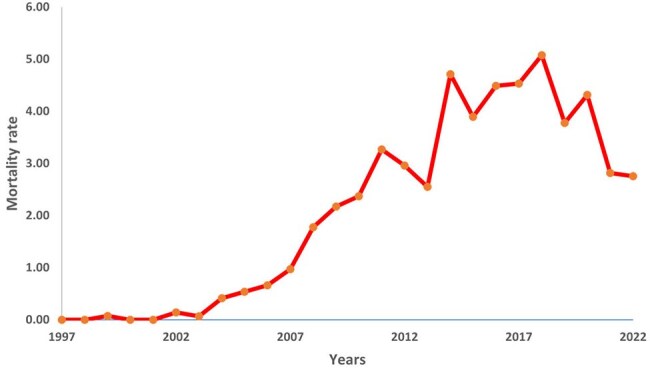
HIV mortality rate per 100,000 population, Sughd Region, Tajikistan, from 1997 to 2022.

**Table 1.**
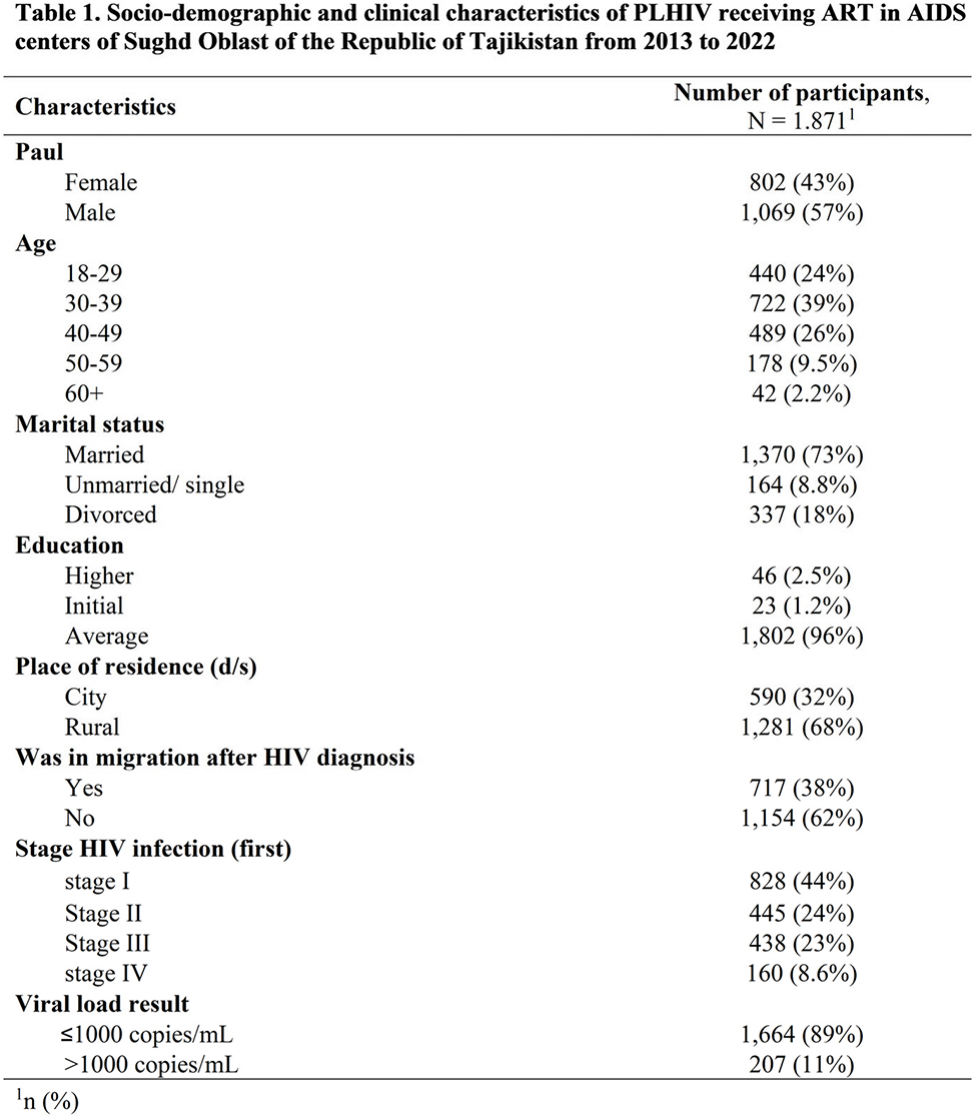
Socio-demographic and clinical characteristics of PLHIV receiving ART in AIDS centers of Sughd Oblast of the Republic of Tajikistan from 2013 to 2022

**Methods:**

We conducted a retrospective cohort study among PLHIV (≥18 years) who received ART for ≥6 months in 2013–2022 in the Sughd region. Data were extracted from the national electronic HIV case registry. Descriptive statistics were used to summarize the characteristics of study participants. Multivariable Poisson regression was used to estimate the adjusted relative risks (aRR) and 95% confidence intervals (CI) associated with HIV mortality.

**Table 2:**
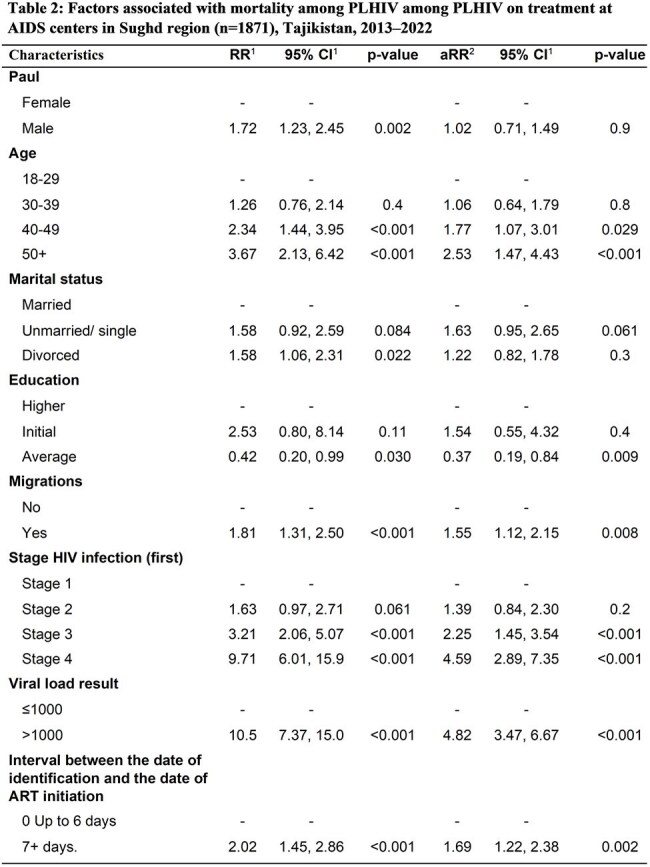
Factors associated with mortality among PLHIV among PLHIV on treatment at AIDS centers in Sughd region (n=1871), Tajikistan, 2013–2022 1 RR = Risk ratio, 2 CI = Confidence Interval 3 aRR = adjusted Risk ratio

**Results:**

A total of 1,871 PLHIV received ART for ≥ 6 months in 2013–2022. Of whom, 57% were men, the mean age was 31 years (standard deviation=9.8), 96% had secondary education, 68% lived in rural areas, and 38% were migrants (Table 1). One-third (32%) were diagnosed with late-stage HIV (23% in stage 3 and 9% in stage 4). Overall, 163 (9%) died (cohort mortality rate of 17.4 deaths per 1,000 person-years). Mortality was associated with age 40–49 years (aRR=1.8, 95% CI=1.1–3.0, p< 0.001) and 50 years vs < 40 (aRR=2.5, 95% CI: 1.4–4.4 p< 0.001) migration after HIV diagnosis vs no migration (RR=1.6, 95% CI=1.1-2.2, p< 0.01), HIV stage 4 (aRR=4.6, 95% CI=2.9–7.3, p< 0.01) and stage 3 vs stage 1 (aRR=2.3, 95% CI: 1.5–3.5, p< 0.01), viral load >1000 copies/mL vs ≤1000 (aRR=4.8, 95% CI=3.5–6.7, p< 0.001), and ART initiation ≥7 days after diagnosis vs < 7 days (aRR=1.7, 95% CI=1.2–2.4, p< 0.001).

**Conclusion:**

The HIV mortality rate is high in the Sughd region. Late initiation was an important risk factor for mortality. Interventions to increase earlier diagnosis and timely initiation of ART, especially among older and migrant PLHIV, may reduce mortality.

**Disclosures:**

All Authors: No reported disclosures

